# Use of CRISPR/Cas9-Based Gene Editing to Simultaneously Mutate Multiple Homologous Genes Required for Pollen Development and Male Fertility in Maize

**DOI:** 10.3390/cells11030439

**Published:** 2022-01-27

**Authors:** Xinze Liu, Shaowei Zhang, Yilin Jiang, Tingwei Yan, Chaowei Fang, Quancan Hou, Suowei Wu, Ke Xie, Xueli An, Xiangyuan Wan

**Affiliations:** 1 Zhongzhi International Institute of Agricultural Biosciences, Shunde Graduate School, Research Center of Biology and Agriculture, University of Science and Technology Beijing (USTB), Beijing 100024, China; b20180388@xs.ustb.edu.cn (X.L.); b20200413@xs.ustb.edu.cn (S.Z.); b20190393@xs.ustb.edu.cn (Y.J.); b20190395@xs.ustb.edu.cn (T.Y.); b20190392@xs.ustb.edu.cn (C.F.); houquancan@ustb.edu.cn (Q.H.); suoweiwu@ustb.edu.cn (S.W.); xieke@ustb.edu.cn (K.X.); 2 Beijing Engineering Laboratory of Main Crop Bio-Tech Breeding, Beijing International Science and Technology Cooperation Base of Bio-Tech Breeding, Beijing Solidwill Sci-Tech Co., Ltd., Beijing 100192, China

**Keywords:** CRISPR/Cas9, gene editing, multiple homologous genes, pollen development, genic male sterility, maize

## Abstract

Male sterility represents an important trait for hybrid breeding and seed production in crops. Although the genes required for male fertility have been widely studied and characterized in many plant species, most of them are single genic male-sterility (GMS) genes. To investigate the role of multiple homologous genes in anther and pollen developments of maize, we established the CRISPR/Cas9-based gene editing method to simultaneously mutate the homologs in several putative GMS gene families. By using the integrated strategies of multi-gene editing vectors, maize genetic transformation, mutation-site analysis of T_0_ and F_1_ plants, and genotyping and phenotyping of F_2_ progenies, we further confirmed gene functions of every member in *ZmTGA9-1/-2/-3* family, and identified the functions of *ZmDFR1*, *ZmDFR2*, *ZmACOS5-1*, and *ZmACOS5-2* in controlling maize male fertility. Single and double homozygous gene mutants of *ZmTGA9-1/-2/-3* did not affect anther and pollen development, while triple homozygous gene mutant resulted in complete male sterility. Two single-gene mutants of *ZmDFR1/2* displayed partial male sterility, but the double-gene mutant showed complete male sterility. Additionally, only the *ZmACOS5-2* single gene was required for anther and pollen development, while *ZmACOS5-1* had no effect on male fertility. Our results show that the CRISPR/Cas9 gene editing system is a highly efficient and convenient tool for identifying multiple homologous GMS genes. These findings enrich GMS genes and mutant resources for breeding of maize GMS lines and promote deep understanding of the gene family underlying pollen development and male fertility in maize.

## 1. Introduction

Maize (*Zea mays* L.) is an important food and feed crop worldwide. As a fast growing C4 plant and a successful crop of heterosis utilization, it provides more than one-half of global calorie consumption [1]. In recent decades, the wide use of hybrid varieties has greatly increased maize yield. Manual or mechanical detasseling is required for maize hybrid seed production to prevent self-pollination. However, detasseling has obvious drawbacks, e.g., it is time-consuming, labor-intensive, expensive, affects plant growth, and reduces the yield of hybrid seeds [2]. Therefore, developing a male-sterility line is critical for producing hybrid maize seed.

Male sterility is a useful agronomy trait for hybrid seed production in crops, but its application in maize is not successful and has suffered from several problems. For example, cytoplasmic male sterility has been used in the production of commercial hybrid maize; however, due to the potential increase of disease susceptibility and unreliable restoration of cytoplasmic male sterility line, it is limited in producing hybrid seeds [3]. Environment sensitive genic male sterility has an unstable fertility risk under variable environments [4]. Genic male sterility (GMS) caused by nuclear genes alone can overcome the disadvantages mentioned above, but it suffers from producing large-scale GMS seeds through self-pollination. Fortunately, with technological advances, some biotechnology-based male-sterility systems with GMS genes have been developed successfully in crops, which includes seed production technology, multi-control sterility, and dominant male sterility in maize [5,6,7]. Thus, the isolation and identification of GMS genes has become a research focus, for developing biotechnology-based male-sterility systems in maize.

Thus far, more than 100 GMS genes have been identified and characterized in plants, most of which are reported to encode transcription factors (TFs) or lipid biosynthesis and transport proteins, including 49 in *Arabidopsis*, 38 in rice, and 24 in maize [2,8,9,10]. GMS genes isolated in maize, compared with *Arabidopsis* and rice, are relatively few. Notably, by combining maize anther RNA-seq and comparative genomics analyses, 62 orthologs of GMS genes reported in other plants have been predicted previously in maize, consisting of 19 TFs, 18 lipid metabolic genes, and 37 genes involved in other processes [2]. Among them, 35 genes correspond to 16 gene families, and the number of paralogs in each gene family is between 2 and 4 [2]. Analysis of 16 fully sequenced genomes in plants, such as maize, rice, and wheat, has shown that the paralogous gene families account for about 72% of protein-coding genes [11]. Therefore, it is possible that multiple homologous genes are required for male fertility in plants. To date, few homologous GMS genes have been identified, since it is relatively difficult to simultaneously mutate multiple homologous genes through spontaneous mutation or traditional mutagenesis methods. Whether members of a gene family have functional redundancy in male fertility requires mutating two or more paralogs, and then evaluating their phenotypic effects of single and multiple mutations. Thus, the simultaneous editing of multiplex genes makes it possible to simultaneously modify the multi-DNA sequences and further explore the functional redundancy of multiple genes [12].

Gene-editing technologies can efficiently introduce precise and predictable gene mutations into plants to obtain desired phenotypes [13,14]; the CRISPR/Cas system is the most popular system due to its specificity, simplicity, flexibility, and versatility [15]. Multiplex gene editing can be achieved with Cas9 nuclease through expressing Cas9 along with multiple gRNAs [16], which has been successfully used to dissect the functions of gene family members with redundant functions in plants [17]. Additionally, the CRISPR/Cpf1 system is a natural, multi-unit system, with a simple short direct repeat (DR)-based unit itself. The Cpf1 nuclease has the ability to process its own CRISPR RNA and can be used to simplify multiplexed genome editing [18]. Although the CRISPR/Cpf1 system has been shown to efficiently introduce multiple-gene mutations into plants, such as maize, rice, and soybean [19,20,21], it still needs to be further optimized because its editing efficiency is lower than that of the CRISPR/Cas9 system.

Bread wheat is allohexaploid and consists of three genomes (A-, B-, and D-) that exhibit extensive functional redundancy, which makes it unusually difficult to obtain the recessive nuclear male sterile mutants through traditional mutagenesis. Using the CRISPR/Cas9 system, some homologous GMS genes have been reported in bread wheat, including *TaMs45*, the ortholog of *Ms45* in maize and *LAP3* in *Arabidopsis* [22], and *TaNP1*, the ortholog of *OsNP1* and *ZmIPE1* [23]. Both *TaMs45* triple mutant and *TaNP1* triple mutant display complete male sterility, but one WT copy of *TaMs45* or *TaNP1* genes is sufficient for maintenance of male fertility. In addition, the CRISPR/Cas9 technology has been successfully utilized to identify homologous TF GMS genes in maize [24]. A *ZmGAMYB* double mutant displayed complete male sterility, but not for the single-gene mutants. Overall, the studies on the multiplex homologous GMS gene by the CRISPR/Cas9 system are relatively few in plants, and the corresponding research method requires further development.

Here, we established an efficient method to simultaneously mutate multiplex homologous GMS genes in maize with the multi-gene editing strategy based on CRISPR/Cas9. The creation and selection processes of different-type mutants of *ZmTGA9-1/-2/-3*, *ZmDFR1/2*, and *ZmACOS5-1/-2* families are described in detail. Finally, by combing genotypic and phenotypic analyses, we elucidated the functions of three multi-gene families on male fertility, which will facilitate understanding the functional redundancy of gene family on anther and pollen development.

## 2. Materials and Methods

### 2.1. Plant Materials and Growth Conditions

Maize inbred line Zheng 58 and hybrid Hi-II seeds were provided by our laboratory. All maize materials were grown in the experimental stations at the University of Science and Technology Beijing under normal cultivation conditions, except maize transgenic plants that were grown in a greenhouse under 16 h of light/8 h of dark at 26 °C.

### 2.2. Characterization of Mutant Phenotypes

A Canon EOS 700D digital camera (Canon, Tokyo, Japan) and a SZX2-ILLB stereomicroscope (Olympus, Tokyo, Japan) were used to take photographs for tassels and anthers, respectively. A BX-53 microscope (Olympus, Tokyo, Japan) was used to assay its mature pollen grains with 1% I_2_-KI solution [25].

### 2.3. Plasmid Construction

For generating the CRISPR/Cas9-mediated multi-gene mutants, the 19-bp fragment targeting the predictive genes were designed on the CRISPR-P 2.0 (http://crispr.hzau.edu.cn/CRISPR2/ accessed on 20 July 2019) and evaluated on a website (http://www.rgenome.net/cas-offinder/ accessed on 20 July 2019), respectively [26]. The vector *pCBC-MT1T2* was used, and two 19-bp fragments were amplified based on the primer pair specific for each gene and introduced into the *BsaI*-digested *pBUE411* vector [27]. The primers are listed in Table S1.

### 2.4. Maize Genetic Transformation

The recombinant plasmids were used for *Agrobacterium tumefaciens*-mediated transformation into maize (Hi-II) [27]. The *Bar* gene was used as a selectable marker for selecting positive transformants by PCR amplification using primers OGF41 and OGF42. The primers are listed in Table S1.

### 2.5. Genotyping Maize Mutant Plants

The cetyltrimethylammonium bromide (CTAB) method was used to extract genomic DNA from the leaves of maize seedlings [28]. PCR amplifications of relevant regions in transgenic plants were accomplished using the specific primers in Table S1. For genotyping plants in T_0_ and F_1_ generations, the PCR products of relevant regions were purified and introduced into the *pEASY-T5 Zero Cloning* vector (TransGen Biotech, Beijing, China) for DNA sequencing. The sequencing chromatograms were meticulously analyzed for exact patterns that might indicate the mutation types, i.e., homozygous, monoallelic, or diallelic mutations. The co-segregating molecular markers were designed according to mutations of target genes (Table S1), as described previously [29]. The PCR products were analyzed in F_2_ generation using polyacrylamide gel electrophoresis.

### 2.6. Statistical Analysis

The segregation of sterility phenotypes was assessed in the F_2_ populations. Goodness of fit to theoretical ratios was evaluated using the chi-square (Χ^2^) test in accordance with methods as described previously [30], i.e., Χ^2^ = 6(|O − E| − 0.5)^2^/E or Χ^2^ = 6(O − E)^2^/E, where E and O are the expected and observed frequencies, respectively.

## 3. Results

### 3.1. ZmTGA9-1, ZmTGA9-2 and ZmTGA9-3 Display Completely Functional Redundancy to Control Anther and Pollen Development in Maize

*AtTGA9* and *AtTGA10* are involved in regulating a common set of genes that contribute to tapetum and anther development [31]. *ZmTGA9-1* (*Zm00001d052543*), *ZmTGA9-2* (*Zm00001d042777*), *ZmTGA9-3* (*Zm00001d012294*), *ZmbZIP87* (*Zm00001d038296*), and *ZmbZIP109* (*Zm00001d023424*) are the orthologs of *TGA9* in *Arabidopsis* (Figure S1(A1)). However, only *ZmTGA9-1*, *ZmTGA9-2*, and *ZmTGA9-3* are expressed during maize anther development based on RNA-seq data of W23 anther (Figure S1(A2)) [32], indicating the potentially critical roles of the three genes in controlling maize male reproduction. Based on co-introduced of two vectors containing Cas9 and sgRNA expression cassettes into maize, our previous research revealed that *ZmTGA9* triple mutant displayed complete male sterility, while single and double mutants of *ZmTGA9-1/-2/-3* showed normal fertility [10]. However, the co-transformation method influences transformation efficiency and especially increases the complexity of molecular identification. To establish a more efficient method for simultaneously editing homologous GMS genes, in this study, we only transferred one CRISPR/Cas9 editing vector into maize to produce novel knockout-mutants of *ZmTGA9-1/-2/-3*, which further confirmed the function of the *ZmTGA9* family on male fertility.

**Figure 1:**
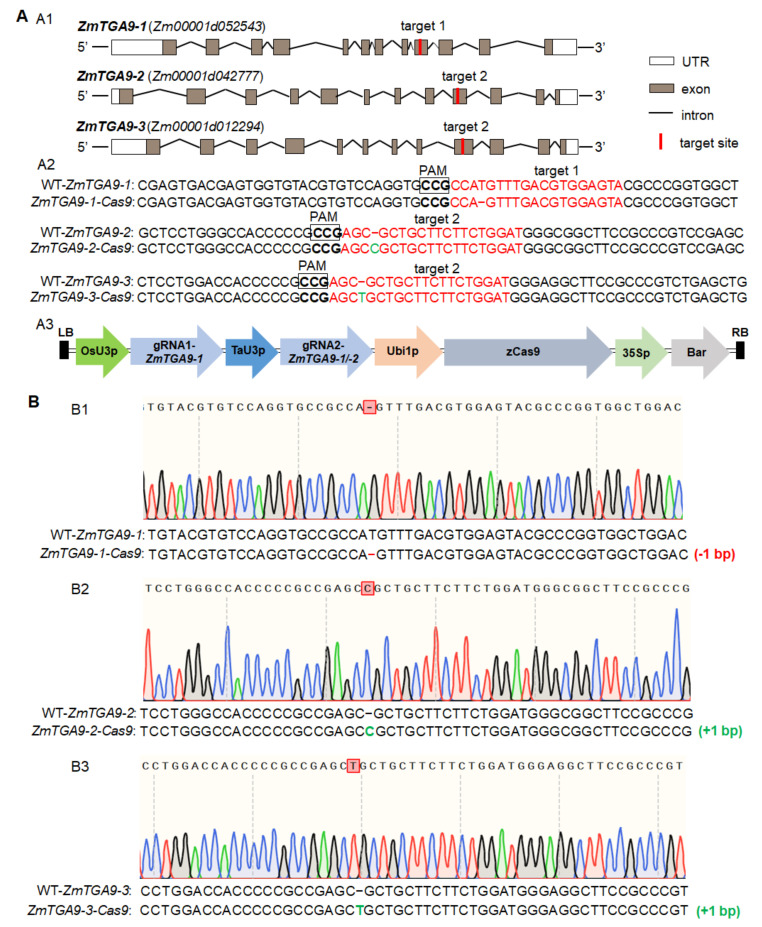
The CRISPR/Cas9 mutagenesis of *ZmTGA9-1*, *ZmTGA9-2*, and *ZmTGA9-3* and the derived mutant sequencing analysis. (**A**) The CRISPR/Cas9 mutagenesis of *ZmTGA9-1*, *ZmTGA9-2*, and *ZmTGA9-3.* (**A1**) Diagram showing the two target sites on the *ZmTGA9-1*, *ZmTGA9-2*, and *ZmTGA9-3*, respectively. (**A2**) Sequence analysis of the target sites on *ZmTGA9-1*, *ZmTGA9-2*, and *ZmTGA9-3* and their mutants. The target sites and protospacer-adjacent motif (PAM) sequences are shown in the antisense strand and highlighted in red and boldface fonts, respectively. Deletions and insertions are indicated by red, short, dashed lines and green fonts, respectively. (**A3**) Diagram illustrating the sgRNA expression cassettes targeting *ZmTGA9-1*, *ZmTGA9-2*, and *ZmTGA9-3* via the dual-sgRNAs CRISPR/Cas9 vector system. (**B**) Identification of mutations in a homozygous *zmtga9-1/-2/-3* triple knockout line by Sanger sequencing. (**B1**) Sanger sequencing result of mutation in the *ZmTGA9-1* gene. (**B2**) Sanger sequencing result of mutation in the *ZmTGA9-2* gene. (**B3**) Sanger sequencing result of mutation in the *ZmTGA9-3* gene. Deletions and insertions are indicated by red, short, dashed lines and green fonts, respectively.

For simultaneously mutating three homologous genes by one gene-editing construct, target 1 was designed in the ninth exon of *ZmTGA9-1*, and target 2 was designed in the ninth exons of *ZmTGA9-2* and *ZmTGA9-3* that have highly similar structures (Figure 1(A1,A2)). One vector containing a Cas9 protein and two sgRNA expression cassettes were constructed (Figure 1(A3)), and then introduced into maize hybrid Hi-II by *Agrobacterium*-mediated transformation to simultaneously edit the three paralogs. Four positive events were obtained in T_0_ plants and used for further mutation analysis and phenotype observation. DNA sequencing of three target sites revealed that up to 84% of T_0_ plants contained mutations in one site at least, while five plants generated from one event were homozygous triple mutants. We identified a homozygous loss-of-function triple mutant of *ZmTGA9-1/-2/-3* with frameshift and truncation mutations from the five plants mentioned above (Figure S2A), including 1-bp deletion in *ZmTGA9-1*, and 1-bp insertion in *ZmTGA9-2* and *ZmTGA9-3*, respectively (Figure 1(A2),B), which is a new allelic mutant differing from *ZmTGA9* triple mutants obtained previously [10]. Since maize T_0_ transgenic plants could not usually produce seeds by self-pollination in the greenhouse, we pollinated the T_0_ plants with pollen grains from maize inbred line Zheng58, and then harvested F_1_ seeds that could be divided into Cas9-positive (transgene) and Cas9-negative (non-transgene) segregation. To obtain stable mutants that eliminated the interference of sgRNA and Cas9 protein, we chose Cas9-negative F_1_ plants having WT genotype from Zheng58 and heritable mutations in three paralogs, to perform self-pollination for generating F_2_ seeds.

**Figure 2:**
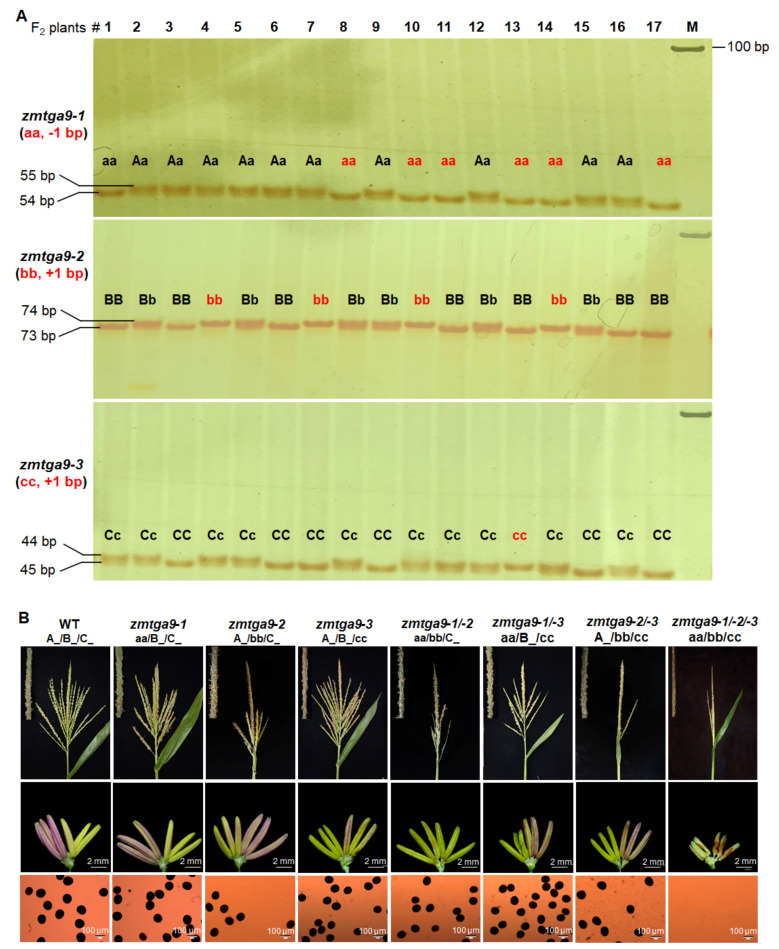
Co-segregating molecular marker genotyping and phenotypic characterization of *ZmTGA9-1/-2/-3-Cas9* mutants in F_2_ population. (**A**) The genotypic identifications of representative *ZmTGA9-1/-2/-3*-*Cas9* F_2_ individuals using developed co-segregating molecular markers. Molecular markers covering 1-bp deletion, 1-bp insertion and 1-bp insertion in the *ZmTGA9-1/-2/-3* genes were detected by using PCR amplification with PAGE, respectively. M, marker. (**B**) Phenotypic analysis of tassels, anthers and pollen grains stained with 1% I_2_-KI solution in WT and the single-, double- and triple-gene homozygous mutants of *ZmTGA9-1/-2/-3*. A and a, B and b, and C and c represent the WT and mutant genotypes of *ZmTGA9-1/-2/-3*, respectively. A_/B_/C_ consists of AA/BB/CC, Aa/BB/CC, AA/Bb/CC, AA/BB/Cc, Aa/Bb/CC, Aa/BB/Cc, AA/Bb/Cc, and Aa/Bb/Cc genotypes. aa/B_/C_ consists of aa/BB/CC, aa/Bb/CC, aa/BB/Cc, and aa/Bb/Cc genotypes. A_/bb/C_ consists of AA/bb/CC, Aa/bb/CC, AA/bb/Cc, and Aa/bb/Cc genotypes. A_/B_/cc consists of AA/BB/cc, Aa/BB/cc, AA/Bb/cc, and Aa/Bb/cc genotypes. aa/bb/C_ consists of aa/bb/CC and aa/bb/Cc genotypes. aa/B_/cc consists of aa/BB/cc and aa/Bb/cc genotypes. A_/bb/cc consists of AA/bb/cc and Aa/bb/cc genotypes.

To detect genotypes of the derived *ZmTGA9-1/-2/-3-Cas9* F_2_ plants, co-segregating molecular markers with the primer pairs covering the corresponding mutations in *ZmTGA9-1*, *ZmTGA9-2*, and *ZmTGA9-3* were firstly developed, respectively (Table S1). Furthermore, the genotypes of 85 F_2_ individuals were identified using PCR amplification together with polyacrylamide gel electrophoresis (PAGE) and classified to eight types: A_/B_C_, aa/B_/C_, A_/bb/C_, A_/B_/cc, aa/bb/C_, aa/B_/cc, A_/bb/cc, and aa/bb/cc (Figure 2A, (Table 1 and Table S2). A further phenotypic analysis showed that only triple homozygous mutant (aa/bb/cc) had shrunken anthers and failed to produce viable pollen grains (Figure 2B), while three single homozygous mutants (aa/B_/C_, A_/bb/C_, and A_/B_/cc) and three double homozygous mutants (aa/bb/C_, aa/B_/cc, and A_/bb/cc) exhibited normal fertility just like WT (A_/B_C) (Figure 2B), indicating that *ZmTGA9-1*, *ZmTGA9-2*, and *ZmTGA9-3* are completely functional redundant. The proportions of normal and aborted pollen grains in WT and single, double, and triple mutants of *ZmTGA9-1/-2/-3* further confirmed the function of the *ZmTGA9* family on male fertility (Figure S3A). In addition, the F_2_ plants displayed an approximate ratio of 63:1 for fertility to sterility (Table 1and Table S2), indicating a recessive three-factor inheritance characteristic of the *zmtga9-1/-2/-3* mutant. Taken together, the roles of three homologous *ZmTGA9* genes in male fertility were further characterized using the efficient CRISPR/Cas9 system.

### 3.2. ZmDFR1 and ZmDFR2 Have Partially Redundant Functions in Controlling Maize Male Fertility

*ZmDFR1* (*Zm00001d031488*) and *ZmDFR2* (*Zm00001d020970*) are the orthologs of GMS genes *OsTKPR1* and *AtTKPR1* (Figure S1) [33,34], and the two genes are all preferentially expressed at anther developmental stages S8 to S9 (Figure S1(B2)). To explore their functions on male fertility in maize, we produced both single- and double-gene mutants of *ZmDFR1/2* via the CRISPR/Cas9 system.

**Figure 3:**
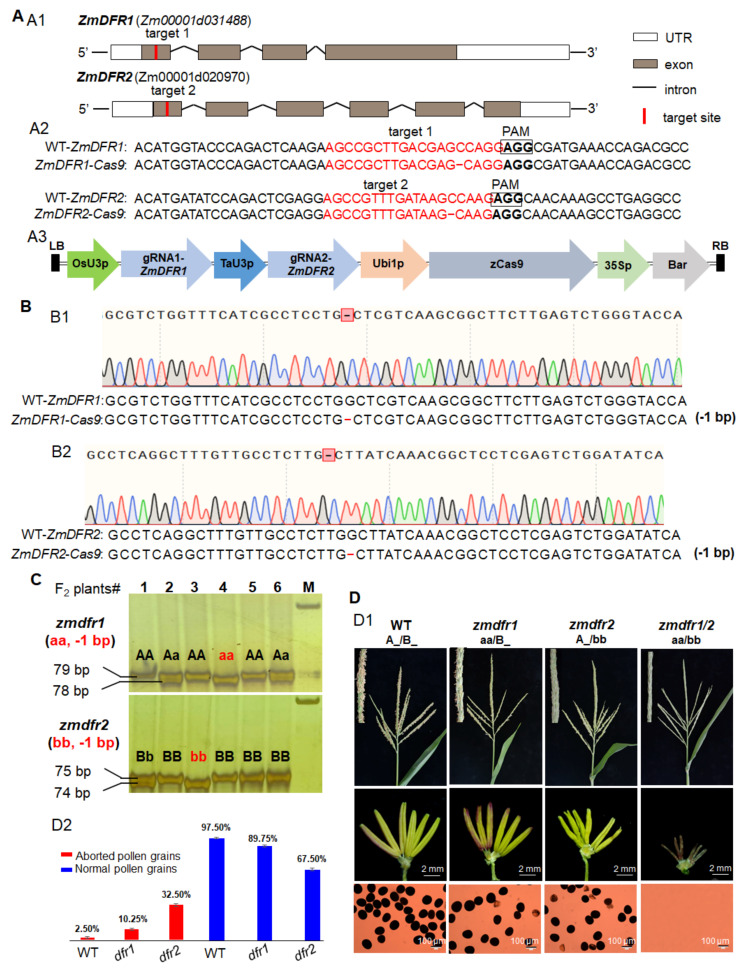
The CRISPR/Cas9 mutagenesis of *ZmDFR1* and *ZmDFR2*, and co-segregating molecular marker genotyping and phenotypic characterization of *ZmDFR1/2-Cas9* mutants in F_2_ population. (**A**) The CRISPR/Cas9 mutagenesis of *ZmDFR1* and *ZmDFR2.* (**A1**) Diagram showing the two target sites on the *ZmDFR1* and *ZmDFR2* genes, respectively. (**A2**) Sequence analysis of the target sites in on *ZmDFR1* and *ZmDFR2* and their mutants. The target sites and PAM sequences are shown in the sense strand and highlighted in red and boldface fonts, respectively. Deletions are indicated by red, short, dashed lines. (**A3**) Diagram illustrating the sgRNA expression cassettes targeting *ZmDFR1* and *ZmDFR2* genes via the dual-sgRNAs CRISPR/Cas9 vector system. (**B**) Identification of mutations in a homozygous *zmdfr1/2* double knockout line by Sanger sequencing. (**B1**) Sanger sequencing result of mutation in *ZmDFR1* gene. (**B2**) Sanger sequencing result of mutation in the *ZmDFR2* gene. Deletions are indicated by red, short, dashed lines. (**C**) The genotypic identifications of representative *ZmDFR1/2*-*Cas9* F_2_ individuals using developed co-segregating molecular markers. Molecular markers covering 1-bp deletion and 1-bp deletion in *ZmDFR1* and *ZmDFR2* genes were detected by using PCR amplification with PAGE, respectively. M, marker. (**D**) Phenotypic characterization of *ZmDFR1/2-Cas9* mutants in F_2_ population. (**D1**) Phenotypic analysis of tassels, anthers and pollen grains stained with 1% I_2_-KI solution in WT and the single- and double-gene homozygous mutants of *ZmDFR1/2*. (**D2**) The proportions of normal and aborted pollen grains measured by staining with 1% I_2_-KI solution in WT and the single-gene mutants of *ZmDFR1* and *ZmDFR2* at stage 13 (*n* = 4220 to 4300). A and a represent the WT and mutant genotypes of *ZmDFR1*, respectively. B and b represent the WT and mutant genotypes of *ZmDFR2*, respectively. A_/B_ consists of AA/BB, Aa/BB, AA/Bb, and Aa/Bb genotypes. aa/B_ consists of aa/BB and aa/Bb genotypes. A_/bb consists of AA/bb and Aa/bb genotypes.

As *ZmDFR* has two paralogs in the maize genome (Figure S1(B1,B3)), target 1 was designed in the first exon of *ZmDFR1* and target 2 was designed in the first exon of *ZmDFR2* (Figure 3(A1,A2)), respectively. One vector simultaneously editing the two paralogs was constructed and transformed into maize immature embryos (Figure 3(A3)), and then two positive events were produced from 94 embryos. To identify the mutation sites of *ZmDFR1/2* in primary T_0_ transgenic plants, PCR products containing the target site fragment were subjected to DNA sequencing. Mutation analysis showed that up to 95% targeted deletions occurred in 14 T_0_ plants from two events on the target sites of *ZmDFR1* and *ZmDFR2*, and double homozygous mutations were observed in 3 T_0_ plants from one event. One double homozygous mutant of *ZmDFR1/2* with 1-bp deletion in *ZmDFR1* and *ZmDFR2* (Figure 3(A2),B), respectively, was obtained from the T_0_ plants. Accordingly, the protein sequences of ZmDFR1 and ZmDFR2 displayed frameshift and truncation mutations (Figure S2B). Similarly, Zheng58 was used as male parent to pollinate the T_0_ plants of *ZmDFR1/2-Cas9* mutant to produce F_1_ generation, and then Cas9-negative F_1_ plants were selected to perform self-pollination to further generate F_2_ seeds.

To facilitate genotyping F_2_ progenies, we developed co-segregating molecular markers with the primer pairs covering the mutation fragments in *ZmDFR1* and *ZmDFR2* (Table S1). The larger and smaller DNA fragment deletions can be detected by PCR amplification together with PAGE, respectively (Figure 3C). Total 156 F_2_ plants were genotyped, including four genotypes: A_/B_, aa/B_, A_/bb and, aa/bb (Figure 3C, Table 1 and Table S3). Phenotypic observation indicated that two single homozygous mutants (aa/B_ and A_/bb) have exerted anthers and display slightly partial male sterility with 10.25% and 32.50% of aborted pollen grains, respectively (Figure 3(D1,D2)), while the double homozygous mutant (aa/bb) exhibits complete male sterility with smaller anthers and without visible pollen grains compared with WT (A_/B_) (Figure 3D). These results showed that the single-gene mutants of *ZmDFR1* and *ZmDFR2* are partially sterile phenotype, while the *ZmDFR1/2* double mutant is a completely male sterile phenotype, indicating *ZmDFR1* and *ZmDFR2* act partially redundant in controlling pollen development and male fertility in maize. The segregation of fertile to completely sterile individuals in the F_2_ population fitted an approximate ratio of 15:1. Thus, the male sterility in *zmdfr1/2* preserved a recessive dual-factor inheritance.

### 3.3. ZmACOS5-1 and ZmACOS5-2 Display No Functional Redundancy in Maize Male Fertility

*ZmACOS5-1* (*Zm00001eb083110*) and *ZmACOS5-2* (*Zm00001eb119050*) are the orthologs of GMS genes *AtACOS5* and *OsACOS12* that play an important role in pollen formation and anther development (Figure S1) [35,36]. In addition, both *ZmACOS5-1* and *ZmACOS5-2* are highly expressed at maize anther developmental stages S8 and S9 (Figure S1(C2)). The roles of *ZmACOS5-1/-2* in controlling male sterility remain unclear in maize. Therefore, we generated a double-gene mutant of *ZmACOS5-1/-2* via the CRISPR/Cas9 system to unveil their functions on maize male fertility.

**Figure 4:**
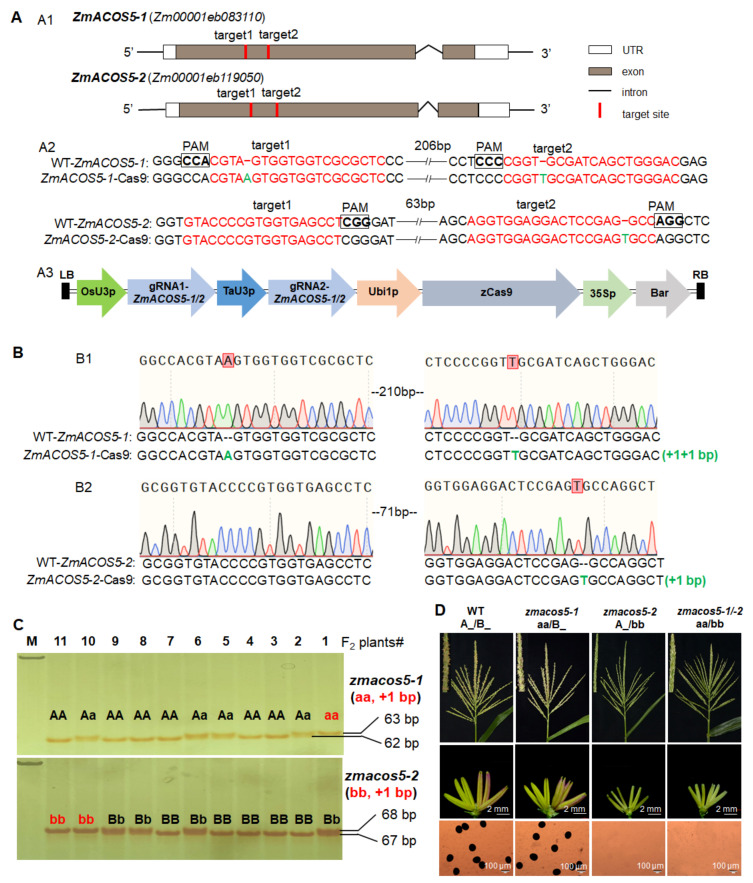
The CRISPR/Cas9 mutagenesis of *ZmACOS5-1* and *ZmACOS5-2*, and co-segregating molecular marker genotyping and phenotypic characterization of *ZmACOS5-1/-2-Cas9* mutants in F_2_ population. (**A**) The CRISPR/Cas9 mutagenesis of *ZmACOS5-1* and *ZmACOS5-2*. (**A1**) Diagram showing the two target sites on the *ZmACOS5-1* and *ZmACOS5-2* genes, respectively. (**A2**) Sequence analysis of the target sites in on *ZmACOS5-1* and *ZmACOS5-2* and their mutants. The target sites and PAM sequences are shown in the sense strand and highlighted in red and boldface fonts, respectively. Insertions are indicated by green fonts. The sequence gap length is shown in the middle of the sequences. (**A3**) Diagram illustrating the sgRNA expression cassettes targeting *ZmACOS5-1* and *ZmACOS5-2* genes via the dual-sgRNAs CRISPR/Cas9 vector system. (**B**) Identification of mutations in a homozygous *zmacos5-1/-2* double knockout line by Sanger sequencing. (**B1**) Sanger sequencing result of mutation in the *ZmACOS5-1* gene. (**B2**) Sanger sequencing result of mutation in the *ZmACOS5-2* gene. Deletions are indicated by red, short, dashed lines. (**C**) The genotypic identifications of representative *ZmACOS5-1/-2*-*Cas9* F_2_ individuals using developed co-segregating molecular markers. Molecular markers covering 1-bp insertion and 1-bp insertion in *ZmACOS5-1* and *ZmACOS5*-*2* genes were detected by using PCR amplification with PAGE, respectively. (**D**) Phenotypic analysis of tassels, anthers and pollen grains stained with 1% I_2_-KI solution in WT and the single- and double-gene homozygous mutants of *ZmACOS5-1/-2*. A and a represent the WT and mutant genotypes of *ZmACOS5-1*, respectively. B and b represent the WT and mutant genotypes of *ZmACOS5-2*, respectively. A_/B_ consists of AA/BB, Aa/BB, AA/Bb, and Aa/Bb genotypes. Aa/B_ consists of aa/BB and aa/Bb genotypes. A_/bb consists of AA/bb and Aa/bb genotypes.

Due to the high sequence similarity in the first exon of *ZmACOS5-1* and *ZmACOS5-2*, two editing targets were designed in the region simultaneously (Figure 4(A1,A2)). One vector containing the two targets was transformed into maize mediated by *Agrobacterium*. After stable transformation of 102 maize immature embryos, DNAs were extracted from young leaves of 21 T_0_ plants produced from three positive events to assess the type and frequency of the mutations. Based on PCR amplification and Sanger sequencing of the target sites, at least 17 plants were detected to have mutations at the designed target 1 or target 2, and the same double-homozygous mutations were observed in 5 T_0_ plants from two events. The double homozygous mutant of *ZmACOS5-1/-2* conferred 2-bp insertion in *ZmACOS5-1* and 1-bp insertion in *ZmACOS5-2*, respectively (Figure 4(A2),B), which resulted in a frameshift and truncation mutations of proteins (Figure S2C). Zheng58 was used as a male line to cross with the *zmacos5-1/-2* mutant to produce F_1_ individuals. To dissect the role of a single gene in male sterility, the F_2_ population was constructed by self-pollination of the Cas9-negative F_1_ plants.

Furthermore, we developed two co-segregating molecular markers with the primer pairs covering mutation fragments in *ZmACOS5-1* (with 1-bp insertion) and *ZmACOS5-2* (with 1-bp insertion), respectively, which were efficiently used to determine the genotypes of 147 F_2_ individuals with PAGE (Figure 4C, Table 1 and Table S4). In the F_2_ population, genotypes consisted of A_/B_, aa/B_, A_/bb_, and aa/bb. The corresponding phenotypic analysis showed that the single homozygous *zmacos5-1* (aa/B_) exhibits normal anther and pollen development, and the proportion of normal pollen grains is just like WT (Figure S3B). In contrast, the single homozygous *zmacos5-2* (A_/bb) and double homozygous *zmacos5-1/-2* (aa/bb) display complete male sterility without pollen grains (Figure 4D). All F_1_ plants from crosses between WT and the double-gene mutant of *ZmACOS5-1/2* showed normal fertility, and the resulting F_2_ plants exhibited a segregation ratio of approximately 3:1 for fertility to sterility, indicating that *zmacos5-1/-2* with complete male sterility is caused by a single recessive mutation of *ZmACOS5-2*. Our results show that *ZmACOS5-1* and *ZmACOS5-2* are not functionally redundant on male fertility in maize, and *ZmACOS5-2* is a GMS gene required for anther and pollen development.

## 4. Discussion

GMS is generally due to a mutation in a nuclear gene; thus, the GMS mutant has obvious advantages, such as non-conditional, recessive, and strictly monogenic inheritance, and is useful for both hybrid breeding and seed production in crops [37]. Although GMS has long been a research focus for many plants, previous studies of the GMS genes have mainly focused on the single gene [25,38,39,40]. The main reason is that it is relatively difficult to simultaneously obtain multi-gene mutations with traditional mutagenesis and map-based cloning methods. In fact, analysis of many plant genomes shows that 72% of protein-coding genes are classified into paralogs [11], suggesting many GMS genes containing multiple homologs are unfortunately missed. Therefore, developing the method of multi-gene precise editing is very important for exploring and discovering more GMS genes with functional redundancy.

The CRISPR/Cas9 system, as a highly efficient and convenient genome editing tool, has been successfully used for simultaneously mutating homologous GMS genes in crops, including bread wheat [22,23], maize [24], and cotton [41]. The corresponding GMS genes include *TaMs45-A/B/D*, *TaMsNP1-A/B/D*, *ZmGAMYB-1/-2*, *GhGPAT12/25*, etc., which is much less than a single GMS gene. To accelerate the discovery of the homologous GMS gene in maize, we produced mutations in each gene of *ZmTGA9-1/-2/-3*, *ZmDFR1/2*, and *ZmACOS5-1/-2* families using two gRNA methods in the CRISPR/Cas9 system (Figure 1, Figure 3 and Figure 4). The triple homozygous mutant, *zmtga9-1/-2/-3*, and double homozygous mutants, *zmdfr1/2* and *zmacos5-1/-2*, all displayed completely male sterility (Figure 2, Figure 3 and Figure 4). The Cas9-free F_1_ plants were selected to generate F_2_ progenies by self-pollination, and then the F_2_ progenies were genotyped by specific, developed molecular markers. Combing the genotyping and phenotyping results, the functions of every member in *ZmTGA9-1/-2/-3*, *ZmDFR1/2*, and *ZmACOS5-1/-2* families on pollen development and male fertility were efficiently defined (Figure 2, Figure 3 and Figure 4 and Tables S2–S4). Our results confirm the powerful capability of the CRISPR/Cas9 system in inducing multiplex mutagenesis simultaneously, further broadening its application in maize functional genomic research. The CRISPR/Cas9 system is a convenient tool to study all members of a GMS gene family, which can avoid combining mutations identified in different homologs through conventional crossing.

In addition, in this study, we only used one gene-editing vector to perform the simultaneous mutation of *ZmTGA9-1*, *ZmTGA9-2*, and *ZmTGA9-3*, which was obviously better than the previously reported co-transformation method of two vectors [42]. During maize transformation, the probability of two T-DNA fragments entering an embryonic cell is generally much lower than that of one T-DNA fragment [42]. Therefore, more embryos, reagents, and drugs are demanded for subsequent processes of genetic transformation. On the other hand, more primers need to be designed for genotyping T_0_ plants; thus, co-transformation takes more time to detect the mutations. Taken together, using the single vector, having dual targets to knock out three *ZmTGA9* simultaneously saves both time and labor.

Different GMS genes with multiple homologs differ in functional redundancy. As transcription factor genes, *ZmTGA9-1*, *ZmTGA9-2*, and *ZmTGA9-3* showed completely redundant functions on the development of anther and pollen. Single and double homozygous mutants in any of the three homologs did not affect pollen development and male fertility, while *ZmTGA9-1/-2/-3* triple mutations resulted in completely male sterility without visible pollen grains (Figure 2, Table S2). Both *ZmDFR1/2* and *ZmACOS5-1/-2* are lipid metabolic genes, and their orthologs in *Arabidopsis* and rice participate in the sporopollenin metabolon [33,34]. Single-gene mutants of *ZmDFR1/2* exhibited slightly partial male-sterility phenotype with exerted anthers, while the double-gene mutant showed complete male sterility (Figure 3, Table S3), suggesting *ZmDFR1* and *ZmDFR2* are partially functional redundant. The knockout mutant of *ZmACOS5-2* showed complete male sterility, while *ZmACOS5-1* displayed normal male fertility (Figure 4, Table S4), indicating that they have no functional redundancy in pollen and anther development. The diversified functions of homologs may be induced by the evolution of these gene families. The detailed molecular regulatory pathways of these newly discovered GMS genes contributing to pollen and anther development in maize need further investigations. Furthermore, the biosynthetic pathways of anther cutin, wax, and sporopollenin is relatively conserved in plants [34,43]. Accordingly, many orthologous lipid metabolic GMS genes show highly conserved functions in anther and pollen development between monocots and dicots [44]. Among the lipid metabolic GMS genes identified previously, five were shared among maize, rice, and Arabidopsis, and ten were shared between rice and *Arabidopsis* [8]. Here, two types (three genes, i.e., *ZmDFR1*/*2* and *ZmACOS5-2*) of lipid metabolic GMS genes in maize, similar to their orthologs (i.e., *AtTKPR1/OsTKPR1* and *AtACOS5/OsACOS12*) in *Arabidopsis* and rice, were found to play relatively conserved roles in controlling anther/pollen development and male fertility. These results support the functional conservation of lipid metabolic GMS genes among different plants.

Collectively, using the reverse genetic strategy (e.g., CRISPR/Cas9) to knock out these candidate homologous GMS genes will enrich the maize GMS mutant and gene resources and, thus, facilitate our understanding of the molecular mechanisms underlying pollen and anther development in maize.

## Figures and Tables

**Table 1:** Genotypic and phenotypic analyses of three knockout lines with simultaneous mutations of two or three homologous genes in F_2_ generation.

F_2_ Lines	Mutation Types	F_2_ Plants									Expected Ratio	Observed Ratio	Χ^2^
*ZmTGA9-1/-2/-3-Cas9*	*tga9-1* (aa): −1 bp *tga9-2* (bb): +1 bp *tga9-3* (cc): +1 bp	Genotypes	A_/B_/C_	aa/B_/C_	A_/bb/C_	A_/B_/cc	aa/bb/C_	aa/B_/cc	A_/bb/cc	aa/bb/cc	27:9:9:9:3:3:3:1	36:15:15:8:2:3:5:1	4.43
		Number	36	15	15	8	2	3	5	1			
		Phenotypes	fertile	fertile	fertile	fertile	fertile	fertile	fertile	complete sterility			
*ZmDFR1/2-Cas9*	*dfr1* (aa): −1 bp *dfr2* (bb): −1 bp	Genotypes	A_/B_	aa/B_	A_/bb	aa/bb					9:3:3:1	11:5:5:1	3.36
		Number	80	35	34	7							
		Phenotypes	fertile	partial sterility	partial sterility	complete sterility							
*ZmACOS5-1/-2-Cas9*	*acos5-1* (aa): +2 bp *acos5-2* (bb): +1 bp	Genotypes	A_/B_	aa/B_	A_/bb	aa/bb					9:3:3:1	13:6:5:1	4.3
		Number	76	36	29	6							
		Phenotypes	fertile	fertile	complete sterility	complete sterility							

## Supplementary Materials

The following supporting information can be downloaded at: https://www.mdpi.com/article/10.3390/cells11030439/s1, Figure S1. Phylogenetic analysis, expression patterns, amino acid sequence alignment of *ZmTGA9-1/-2/-3*, *ZmDFR1/2* and *ZmACOS5-1/-2* families and their putative orthologues or paralogues; Figure S2. Alignment of the amino acid sequences of *ZmTGA9-1/-2/-3*, *ZmDFR1/2*, *ZmACOS5-1/-2*, and their corresponding mutants. Only the sequences flanking the mutations are shown; Figure S3. Pollen characteriatics analysis of *ZmTGA9-1/-2/-3* and *ZmACOS5-1/-2* mutants; Table S1: Primers used in this study; Table S2: Genotypes and phenotypes of all F_2_ plants derived from *ZmTGA9-1/-2/-3-Cas9* line; Table S3: Genotypes and phenotypes of all F_2_ plants derived from *ZmDFR1/2-Cas9* line; Table S4: Geno-types and phenotypes of all F_2_ plants derived from *ZmACOS5-1/-2-Cas9* line.

## Abbreviations

Cas	CRISPR-associated protein
CRISPR	clustered regularly interspaced short palindromic repeats
GMS	genic male sterility
PAGE	polyacrylamide gel electrophoresis
TFs	transcription factors
WT	wild type
