# Supplementary material for: Use of CRISPR/Cas9-Based Gene Editing to Simultaneously Mutate Multiple Homologous Genes Required for Pollen Development and Male Fertility in Maize
Source: Cells. 2022 Jan 27;11(3):439. doi: 10.3390/cells11030439 (PMC8834288; doi:10.3390/cells11030439)
Supplement: Supplementary file 1 [file cells-11-00439-s001.zip › cells-1520959-supplementary/Supplementary Figures -20220117.pdf]

cells MDPI

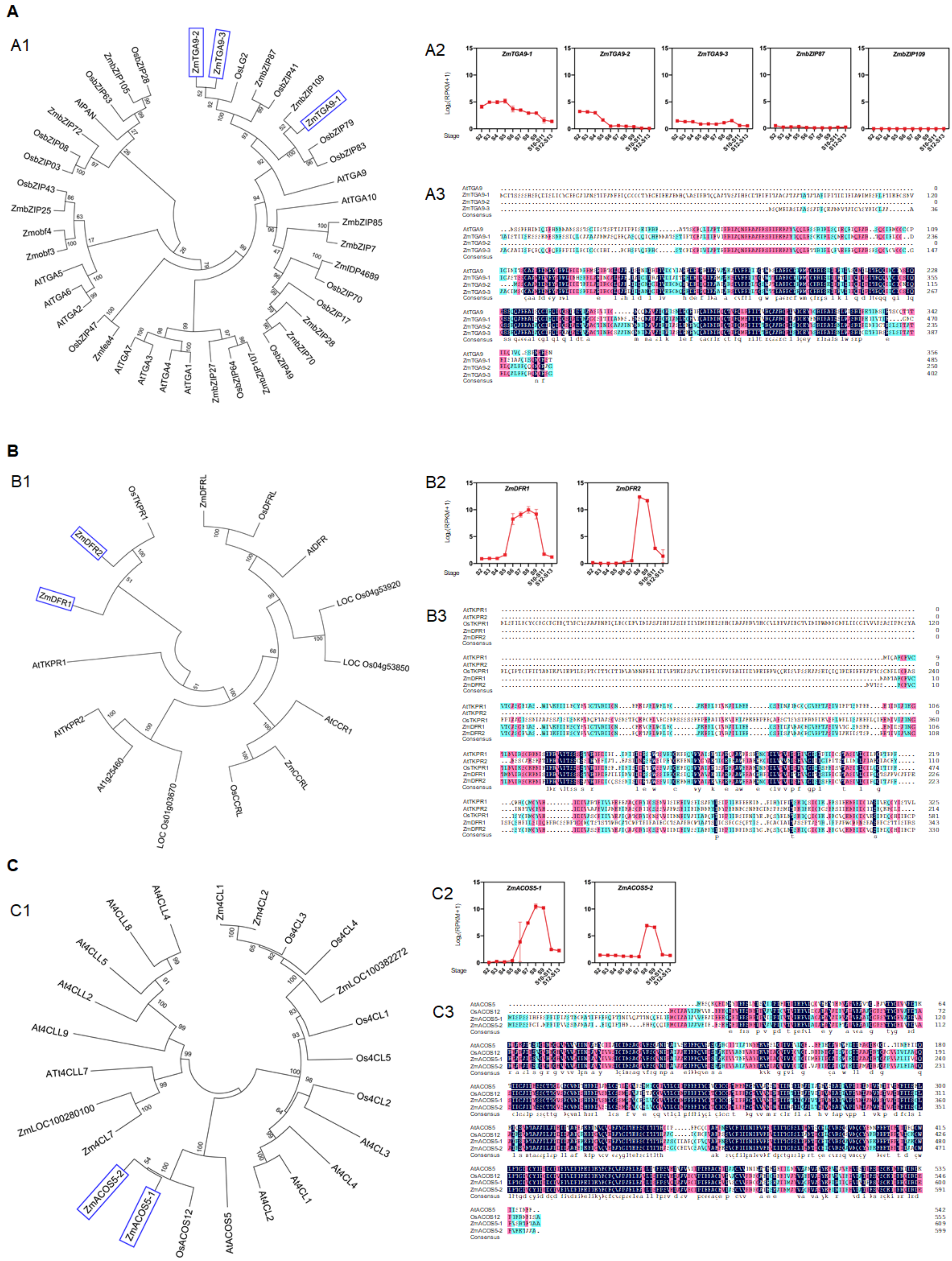
A
A1
ZmTGA9-2
ZmTGA9-3
OsLG2
ZmbZIP87
OsbZIP41
ZmbZIP109
ZmTGA9-1
OsbZIP79
OsbZIP83
AtTGA9
AtTGA10
ZmbZIP85
ZmbZIP7
ZmDP4689
OsbZIP70
OsbZIP17
ZmbZIP28
OsbZIP70
OsbZIP49
ZmbZIP107
OsbZIP64
ZmbZIP27
AtTGA1
AtTGA4
AtTGA3
AtTGA7
Zmfea4
OsbZIP47
AtTGA2
AtTGA6
AtTGA5
Zmobf3
Zmobf4
ZmbZIP25
OsbZIP43
OsbZIP03
OsbZIP08
ZmbZIP72
AtPAN
OsbZIP63
ZmbZIP105
OsbZIP28
A2
ZmTGA9-1
ZmTGA9-2
ZmTGA9-3
ZmbZIP87
ZmbZIP109
Log2(RPKM+1)
Stage
A3
AtTGA9
ZmTGA9-1
ZmTGA9-2
ZmTGA9-3
Consensus
B
B1
OsTKPR1
ZmDFRL
OsDFRL
AtDFR
ZmDFR2
ZmDFR1
LOC Os04g53920
LOC Os04g53850
AtTKPR1
AtCCR1
ZmCCRL
OsCCRL
LOC Os01g03670
At1g25460
AtTKPR2
B2
ZmDFR1
ZmDFR2
Log2(RPKM+1)
Stage
B3
AtTKPR1
AtTKPR2
OsTKPR1
ZmDFR1
ZmDFR2
Consensus
C
C1
At4CLL4
At4CLL8
At4CLL5
At4CLL2
At4CLL9
ATt4CLL7
ZmLOC100280100
Zm4CL7
ZmACOS5-2
ZmACOS5-1
OsACOS12
AtACOS5
Zm4CL1
Zm4CL2
Os4CL3
Os4CL4
ZmLOC100382272
Os4CL1
Os4CL5
Os4CL2
At4CL3
At4CL4
At4CL1
At4CL2
C2
ZmACOS5-1
ZmACOS5-2
Log2(RPKM+1)
Stage
C3
AtACOS5
OsACOS12
ZmACOS5-1
ZmACOS5-2
Consensus

Figure S1. Phylogenetic analysis, expression patterns, amino acid sequence alignment of *ZmTGA9-1/-2/-3*, *ZmDFR1/2* and *ZmACOS5-1/-2* families and their putative orthologues or paralogues. (**A**) Phylogenetic analysis, expression patterns, amino acid sequence alignment of *ZmTGA9-1/-2/-3* family and its putative orthologues or paralogues. (**A1**) Phylogenetic tree of the *bZIP* super family members in *Arabidopsis* (At), rice (Os), and maize (Zm). A neighbour-joining tree displayed the evolutionary relationships of *bZIP* members in the three species. *ZmTGA9-1/-2/-3*, *ZmbZIP87* and *ZmbZIP109* are all the orthologs of *TGA9* in *Arabidopsis.* (**A2**) Expression patterns of five *ZmTGA9* paralogues genes were showed based on RNA-seq data of W23 anther during ten anther developmental stages (S2 to S12). (**A3**) The amino acid sequence alignment of ZmTGA9-1/-2/-3 to their ortholog AtTGA9. (**B**) Phylogenetic analysis, expression patterns, amino acid sequence alignment of *ZmDFR1/2* family and its putative orthologues or paralogues. (**B1**) Phylogenetic tree of the *DFR* family members in *Arabidopsis* (At), rice (Os), and maize (Zm). A neighbour-joining tree displayed the evolutionary relationships of *DFR* members in the three species. *ZmDFR1* and *ZmDFR2* are the orthologs of *AtTKPR1* and *OsTKPR1*. (**B2**) Expression patterns of *ZmDFR1* and *ZmDFR2* were showed based on RNA-seq data of W23 anther during ten anther developmental stages (S2 to S12). (**B3**) The amino acid sequence alignment of ZmDFR1/2 to their orthologs *AtTKPR1* and OsTKPR1. (**C**) Phylogenetic analysis, expression patterns, amino acid sequence alignment of *ZmACOS5-1/-2* family and its putative orthologues or paralogues (**C1**) Phylogenetic tree of the *ACOS* family members in *Arabidopsis* (At), rice (Os), and maize (Zm). A neighbour-joining tree displayed the evolutionary relationships of *ACOS* members in the three species. *ZmACOS5-1* and *ZmACOS5-2* are the orthologs of *AtACOS5* and *OsACOS12*. (**C2**) Expression patterns of *ZmACOS5-1* and *ZmACOS5-2* were showed based on RNA-seq data of W23 anther during ten anther developmental stages (S2 to S12). (**C3**) The amino acid sequence alignment of ZmACOS5-1/-2 to their orthologs AtACOS5 and OsACOS12. Phylogenetic tree is constructed using MEGA6. Bootstrap values (1000 replicates) are given on branches. The amino acid sequences are aligned using DNAMAN 7.0, identity amino acids are shaded dark blue and different amino acids are light blue or pink.

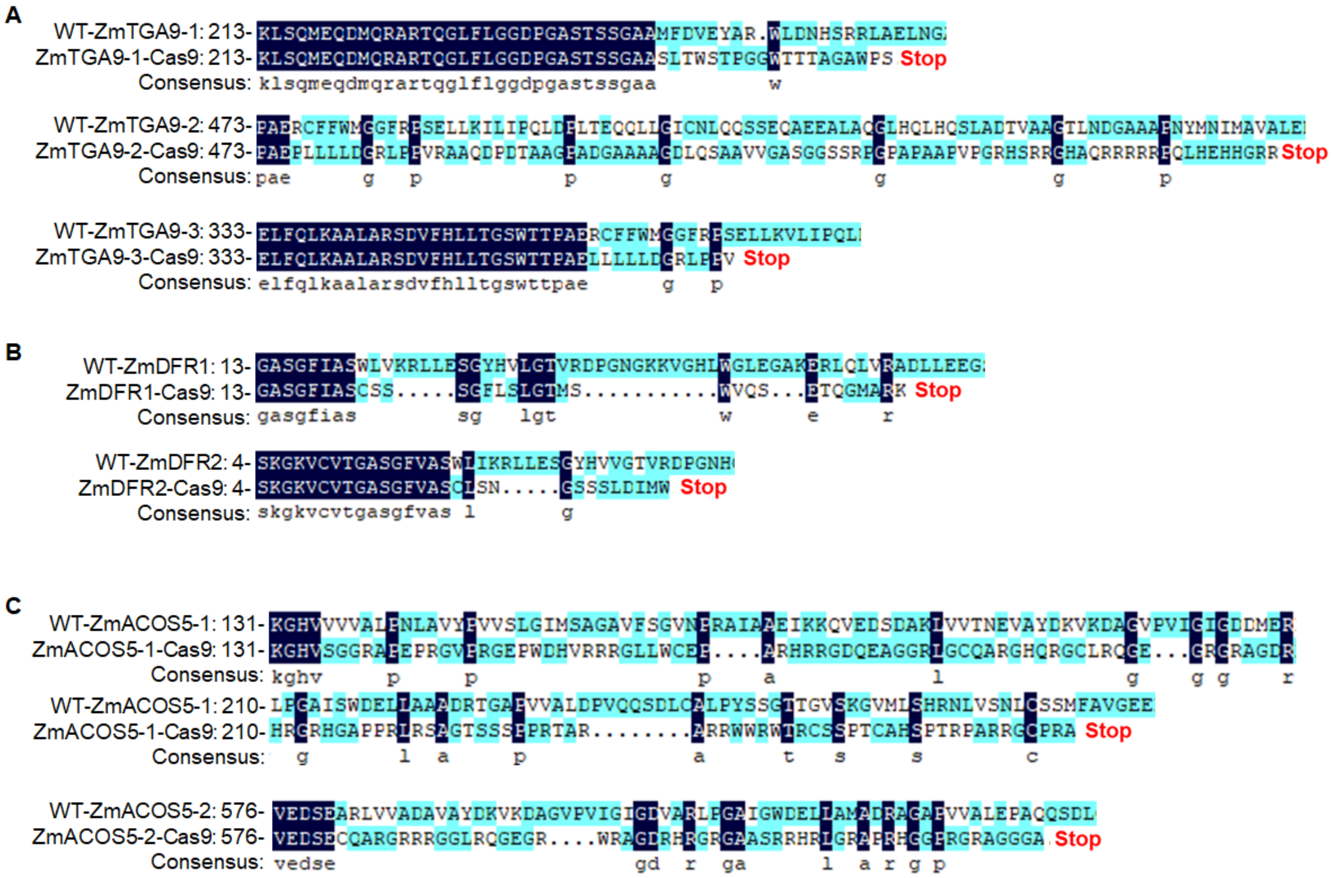

Figure S2. Alignment of the amino acid sequences of ZmTGA9-1/-2/-3, ZmDFR1/2, ZmACOS5-1/-2, and their corresponding mutants. Only the sequences flanking the mutations are shown. (**A**) Alignment of the amino acid sequences of ZmTGA9-1 and ZmTGA9-1-Cas9, ZmTGA9-2 and ZmTGA9-2-Cas9, and ZmTGA9-3 and ZmTGA9-3-Cas9. (**B**) Alignment of the amino acid sequences of ZmDFR1 and ZmDFR1-Cas9 and ZmDFR2 and ZmDFR2-Cas9. (**C**) Alignment of the amino acid sequences of ZmACOS5-1 and ZmACOS5-1-Cas9 and ZmACOS5-2 and ZmACOS5-2-Cas9.

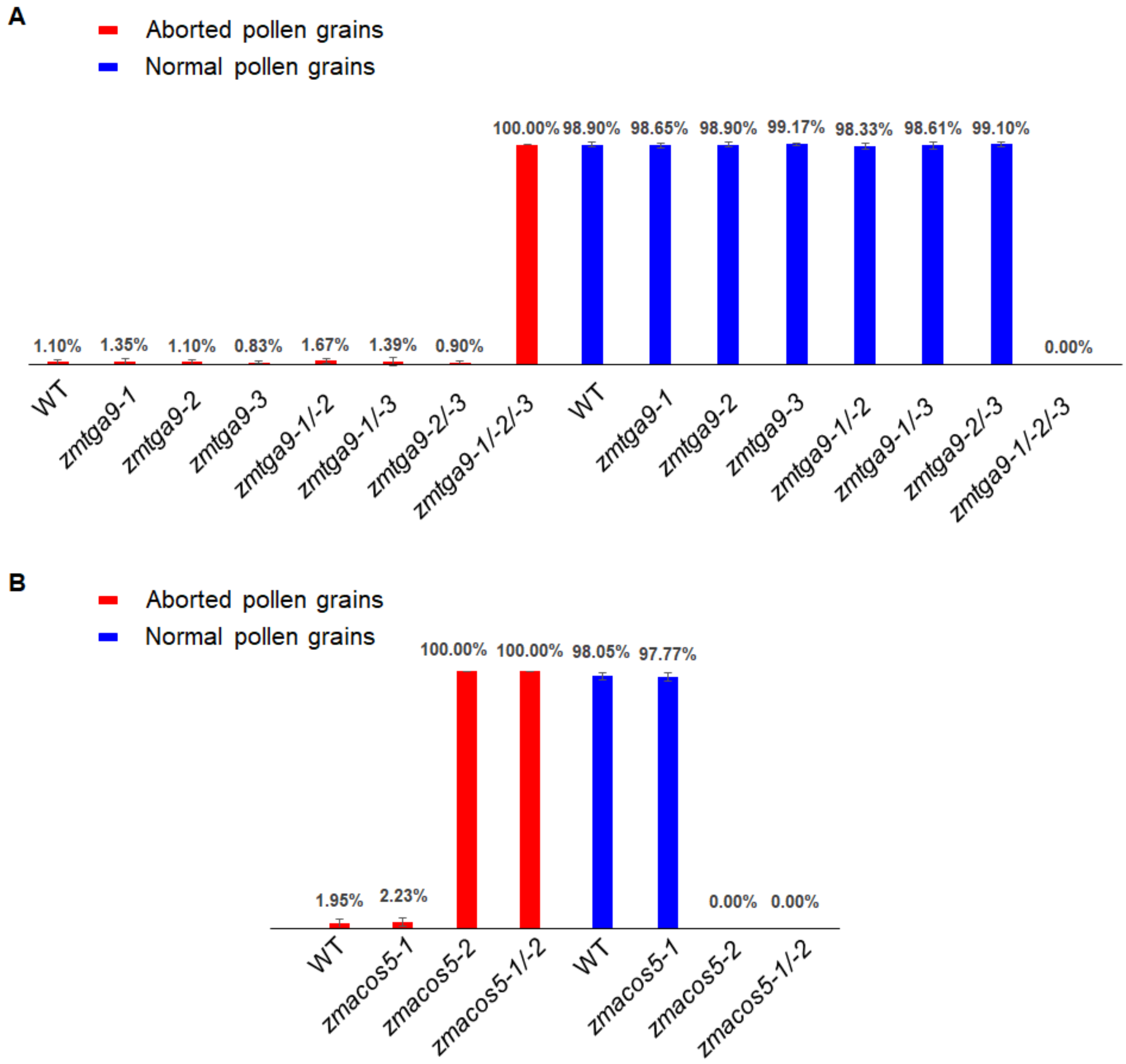

Figure S3. Pollen characteriatics analysis of *ZmTGA9-1*/*-2*/*-3* and *ZmACOS5-1*/*-2* mutants. (**A**) The proportions of normal and aborted pollen grains measured by staining with 1% I_2-KI solution in WT and the single-, double-, and triple-gene mutants of *ZmTGA9-1*/*-2*/*-3* at stage13 (n = 6408 to 6600). (**B**) The proportions of normal and aborted pollen grains in WT and the single- and double-gene mutants of *ZmACOS5-1*/*-2* at stage13 (n = 4400 to 4530).
